# ﻿New species of the genus *Molanna* Curtis, 1834 (Trichoptera, Molannidae) in China inferred from morphology and DNA barcodes

**DOI:** 10.3897/zookeys.1112.84475

**Published:** 2022-07-14

**Authors:** Xin-yu Ge, Lang Peng, Jie Du, Chang-hai Sun, Bei-xin Wang

**Affiliations:** 1 Department of Entomology, College of Plant Protection, Nanjing Agricultural University, 210095, Nanjing, China Nanjing Agricultural University Nanjing China; 2 Jiuzhaigou Administration Bureau, 623402, Jiuzhaigou County, Aba Prefecture, Sichuan Province, China Jiuzhaigou Administration Bureau Sichuan China

**Keywords:** Caddisflies, COI sequence, integrative taxonomy, morphology, Oriental region, wing venation

## Abstract

The male adult of *Molannatruncata* Ge, Peng & Sun **sp. nov.** is described and illustrated based on material collected in Si-chuan, China. It could be diagnosed by the subtriangular superior appendages when viewed dorsally, and by the mesal appendages each having a slender thorn and inferior appendages with a tiny inner process. Based on morphology of genitalia, we provide a dichotomous key to adult males of *Molanna* from the Oriental region. The DNA barcodes (partial mtCOI sequences) of *M.truncata***sp. nov.** are generated and compared with existing sequences of *Molanna* species from Oriental and Palearctic regions. The mean intraspecific divergence of *Molanna* was 1.58% with a maximum of 8.50% in *M.moesta*. The Automatic Barcode Gap Discovery (ABGD) analysis of *Molanna* inferred 9 OTUs and thresholds of interspecific divergence of 10%. Divergence of *M.truncata***sp. nov.** haplotypes from all other *Molanna* haplotypes ranged from 10.1% to 18%. We discuss distribution and potential groups of species within the Oriental *Molanna* species based on morphology.

## ﻿Introduction

Molannidae Wallengren, 1891 is a small family of Holarctic and Oriental biogeographic regions, with two genera, *Molanna* Curtis, 1834 and *Molannodes* McLachlan, 1866 (Morse 2021). Curtis (1834) erected the genus *Molanna* with *Molannaangustata* Curtis, 1834 as the type species by original designation. At present, the genus includes 25 extant species and three fossil species worldwide (Morse 2021). Among them, 12 species were reported from the Oriental region, six were restricted to the Nearctic region, four from the Palaearctic region and two from the East Palaearctic region; only *M.moesta* Banks, 1906 occurs in both the East Palaearctic and Oriental regions (Banks 1906; Malicky and Chantaramongkol 1989; Nebois 1993; Malicky 1994; Ito 2006). At present, three species were reported from China, among them, *M.moesta*, distributed from northeast to southwest in China (i.e. Hei-long-jiang, Jiang-xi, Guang-dong, Si-chuan, Gui-zhou and Yun-nan), while *M.kunmingensis* Hwang, 1957 and *M.xiaguana* Malicky, 1994 are reported from the Yunnan Province exclusively (Yang et al. 2016).

Adults of *Molanna* live around lakes or running waters and are easy to recognize because in repose the adults look like short branch segments (Schmid 1998). Larvae mostly occur in lakes or slower currents of streams, and inhabit sandy substrates (Wiggins 1996). Larvae of *Molannaflavicornis* Banks, 1914 were found to inhabit the profundal zone (up to 20 m deep; Neave 1933).

DNA barcodes, the 658 bp fragment of the mitochondrial gene cytochrome c oxidase I unit (COI), has provided important evidence to confirm new species and has proved to be useful for association between larvae and adults (Hebert et al. 2003; Zhou et al. 2007; Xu et al. 2018; Hu et al. 2019; Ge et al. 2020). But existing DNA barcodes of *Molanna* species from China are very few, and only one barcode has, to date, been recorded in Barcode of Life Data Systems (BOLD) (Ratnasingham and Hebert 2013). This lack of barcode resources greatly restricts accurate monitoring of *Molanna* species for metabarcoding of environmental DNA (Lin et al. 2021).

In this study, we describe a new Chinese species of *Molanna* and provide DNA barcodes of this species. The variation in male hind wing venation and DNA barcodes among species from the Palaearctic and Oriental regions are discussed. Finally, a key and map with distribution to the known adults of *Molanna* from the Oriental region are given.

## ﻿Materials and methods

### ﻿Sample collection

Adult specimens were collected into 95% alcohol using pan traps with 15-W ultraviolet light tubes in the Jiu-zhai-gou County, Si-chuan Province, PR China, during July. The specimens were then sorted and stored in 95% alcohol at -20 °C temperature.

### ﻿Morphological study

The methods used for preparation of genitalia followed Peng et al. (2020). For wing venation studies, Right wings were cut from the body, mounted in a microscope slide with glycerin and covered with a coverslip to ensure that the wings were fully flattened. Wing and genitalia structures were traced in pencil using a Nikon Eclipse 80i microscope and an Olympus SZX10 stereomicroscope equipped with a camera lucida. Pencil drawings were scanned with an Epson Perfection (V30 SE) scanner, then placed as templates in Adobe Photoshop (Version: 13.0) and inked digitally to produce illustrations. The illustrations were then arranged using Adobe Photoshop (version 13.0). Genitalia, wings and the remainder of each specimen were stored in a microvial in 95% alcohol. Type and voucher specimens were deposited at the Insect Collection, Nanjing Agricultural University (NJAU), Nanjing, Jiangsu Province, PR China.

### ﻿Terminology

The terminology for male genitalia follows those of Wiggins (1968) and Ito (2006). The terminology for wing venation follows that of Schmid (1998).

### ﻿DNA analysis

The right hindleg of two adults was removed for genomic DNA extractions. DNA extraction, PCR amplification, fragment sequencing, and analysis followed the procedures of Xu et al. (2018). The universal primers LCO1490 and HCO2198 (Folmer et al. 1994) were used to amplify the 658 bp fragment of the mitochondrial (mt) cytochrome c oxidase I unit (COI). Raw sequences were assembled and edited in Sequencher 4.5 (Gene Codes Corporation, Ann Arbor, Michigan, USA). Neighbor-joining (NJ) tree of eight species within the genus *Molanna* was constructed using MegaX (Kumar et al. 2018), with the following parameters: Kimura 2-parameter substitution model (K2P), pairwise gap deletion, and others as defaults. The same software was used to calculate the K2P corrected *p*-distance of the 658 bp COI fragment among all *Molanna* sequences available (Table 1). COI sequences of *Molanna* species were also applied to the Automatic Barcode Gap Discovery (ABGD) tool (Puillandre et al. 2012) to compare the operational taxonomic unit (OUT) number with the Barcode Index Numbers (BINs). COI sequences of new species were uploaded to GenBank. Accession numbers of the analyzed *Molanna* specimens are shown in Table 1.

**Table 1.** Kimura 2-parameter pairwise genetic distances based on COI barcodes of *Molanna* from the Palaearctic and Oriental regions.

Species	Country	GenBank accessions	*M.angustata*	*M.angustata*	*M.angustata*	*M.angustata*	*M.angustata*	*M.oglamar*	*M. sp.*	*M.nigra*	*M.nigra*	*M.nigra*	*M.nigra*	*M.albicans*	*M.albicans*	*M.albicans*	*M.albicans*	*M.albicans*	*M. XZ sp.*	*M.moesta*	*M.moesta*	*M.moesta*	*M.nervosa*	*M.nervosa*	*M.albicans*	*M.albicans*	*M.albicans*	*M.moesta*	*M.moesta*	*M.moesta*	*M.truncata* sp. nov.	*M.truncata* sp.nov. 1	*M.moesta*	*M.moesta*
*M.angustata*	Germany	GU713189																																
*M.angustata*	Germany	CKX291340	0.003																															
*M.angustata*	Norway	KX293662	0.002	0.005																														
*M.angustata*	Norway	KX295460	0.016	0.020	0.015																													
*M.angustata*	Norway	KX104560	0.005	0.008	0.003	0.018																												
*M.oglamar*	Thailand	KX295021	0.170	0.174	0.168	0.168	0.168																											
*M.* sp.	Japan	LC619232	0.168	0.168	0.168	0.168	0.168	0.167																										
*M.nigra*	Germany	HM422029	0.147	0.143	0.149	0.157	0.147	0.130	0.138																									
*M.nigra*	Estonia	KX291834	0.143	0.139	0.145	0.153	0.145	0.126	0.134	0.006																								
*M.nigra*	Finland	KX143012	0.143	0.143	0.145	0.153	0.145	0.126	0.134	0.008	0.005																							
*M.nigra*	Finland	KX291651	0.147	0.143	0.149	0.157	0.149	0.130	0.136	0.008	0.005	0.003																						
*M.albicans*	Norway	KX103592	0.160	0.156	0.158	0.160	0.162	0.165	0.184	0.176	0.169	0.174	0.174																					
*M.albicans*	Norway	KX105019	0.160	0.156	0.158	0.160	0.162	0.165	0.184	0.176	0.169	0.174	0.174	0.000																				
*M.albicans*	Mongolia	KX103901	0.156	0.152	0.154	0.156	0.158	0.155	0.182	0.173	0.169	0.174	0.174	0.013	0.013																			
*M.albicans*	Mongolia	KX104075	0.156	0.152	0.154	0.156	0.158	0.155	0.182	0.173	0.169	0.174	0.174	0.013	0.013	0.000																		
*M.albicans*	Mongolia	KX106945	0.156	0.152	0.154	0.156	0.158	0.155	0.182	0.173	0.169	0.174	0.174	0.013	0.013	0.000	0.000																	
*M.* XZ.sp.	China	KX102865	0.156	0.156	0.154	0.158	0.154	0.125	0.128	0.113	0.108	0.108	0.112	0.148	0.148	0.146	0.146	0.146																
*M.moesta*	Japan	KX107440	0.166	0.170	0.166	0.166	0.170	0.171	0.035	0.154	0.150	0.150	0.152	0.186	0.186	0.184	0.184	0.184	0.148															
*M.moesta*	Japan	KX103332	0.153	0.158	0.153	0.158	0.158	0.175	0.031	0.150	0.146	0.146	0.148	0.191	0.191	0.193	0.193	0.193	0.148	0.016														
*M.moesta*	Japan	KX105642	0.166	0.170	0.166	0.166	0.170	0.171	0.035	0.154	0.150	0.150	0.152	0.186	0.186	0.184	0.184	0.184	0.148	0.000	0.016													
*M.nervosa*	Japan	KX103890	0.160	0.164	0.158	0.166	0.158	0.131	0.159	0.161	0.157	0.157	0.157	0.178	0.178	0.165	0.165	0.165	0.116	0.170	0.170	0.170												
*M.nervosa*	Japan	KX105001	0.156	0.160	0.154	0.162	0.154	0.127	0.159	0.157	0.153	0.153	0.153	0.174	0.174	0.161	0.161	0.161	0.112	0.170	0.170	0.170	0.003											
*M.albicans*	Mongolia	KX106945	0.156	0.152	0.154	0.156	0.158	0.155	0.182	0.173	0.169	0.174	0.174	0.013	0.013	0.000	0.000	0.000	0.146	0.184	0.193	0.184	0.165	0.161										
*M.albicans*	Mongolia	KX104075	0.156	0.152	0.154	0.156	0.158	0.155	0.182	0.173	0.169	0.174	0.174	0.013	0.013	0.000	0.000	0.000	0.146	0.184	0.193	0.184	0.165	0.161	0.000									
*M.albicans*	Mongolia	KX103901	0.156	0.152	0.154	0.156	0.158	0.155	0.182	0.173	0.169	0.174	0.174	0.013	0.013	0.000	0.000	0.000	0.146	0.184	0.193	0.184	0.165	0.161	0.000	0.000								
*M.moesta*	Russia	KX291805	0.176	0.181	0.176	0.176	0.181	0.159	0.036	0.142	0.138	0.138	0.140	0.193	0.193	0.186	0.186	0.186	0.134	0.042	0.042	0.042	0.153	0.153	0.186	0.186	0.186							
*M.moesta*	Russia	KX295053	0.174	0.178	0.174	0.174	0.178	0.161	0.033	0.144	0.140	0.140	0.142	0.195	0.195	0.188	0.188	0.188	0.136	0.042	0.038	0.042	0.155	0.155	0.188	0.188	0.188	0.003						
*M.moesta*	Russia	KX292654	0.178	0.183	0.178	0.178	0.183	0.161	0.036	0.144	0.140	0.140	0.142	0.195	0.195	0.188	0.188	0.188	0.132	0.042	0.042	0.042	0.155	0.155	0.188	0.188	0.188	0.003	0.003					
*M.truncata* sp. nov.	China	ON437539	0.176	0.180	0.174	0.176	0.176	0.127	0.158	0.138	0.135	0.133	0.137	0.179	0.179	0.170	0.170	0.170	0.101	0.168	0.173	0.168	0.127	0.127	0.170	0.170	0.170	0.156	0.158	0.158				
*M.truncata* sp. nov.	China	ON437540	0.176	0.180	0.174	0.176	0.176	0.127	0.158	0.138	0.135	0.133	0.137	0.179	0.179	0.170	0.170	0.170	0.101	0.168	0.173	0.168	0.127	0.127	0.170	0.170	0.170	0.156	0.158	0.158	0.000			
*M.moesta*	Laos	HQ958937	0.163	0.168	0.163	0.163	0.168	0.159	0.070	0.146	0.141	0.141	0.143	0.176	0.176	0.169	0.169	0.169	0.128	0.085	0.083	0.085	0.151	0.147	0.169	0.169	0.169	0.070	0.070	0.070	0.144	0.144		
*M.moesta*	Laos	KX291103	0.162	0.166	0.162	0.162	0.166	0.163	0.066	0.149	0.145	0.145	0.147	0.178	0.178	0.171	0.171	0.171	0.132	0.085	0.081	0.085	0.153	0.149	0.171	0.171	0.171	0.070	0.066	0.070	0.148	0.148	0.003	

## ﻿Results

### ﻿Taxonomy

#### ﻿ Molanna truncata

Taxon classificationAnimaliaTrichopteraMolannidae

Ge, Peng & Sun sp. nov.

17E539CA-4460-5495-89E8-F2E9A2D96ACC

https://zoobank.org/82EBA03B-56EC-42F7-83E4-3CD0F8D4D3BF

Fig. 1A–F

##### Type material.

***Holotype***: 1♂, P.R. China, Si-chuan Province, Aba Prefecture, Jiu-zhaigou County, Jiu-zhaigou National Nature Reserve, Xi-niu-hai (Fig. 2), 33°11'42"N, 103°53'46"E, alt. 2348 m, 7 VII 2019, leg. X.Y. Ge & Y. Wang (NJAU). ***Paratypes***: 2 ♂, same data as holotype (NJAU).

**Figure 1.**
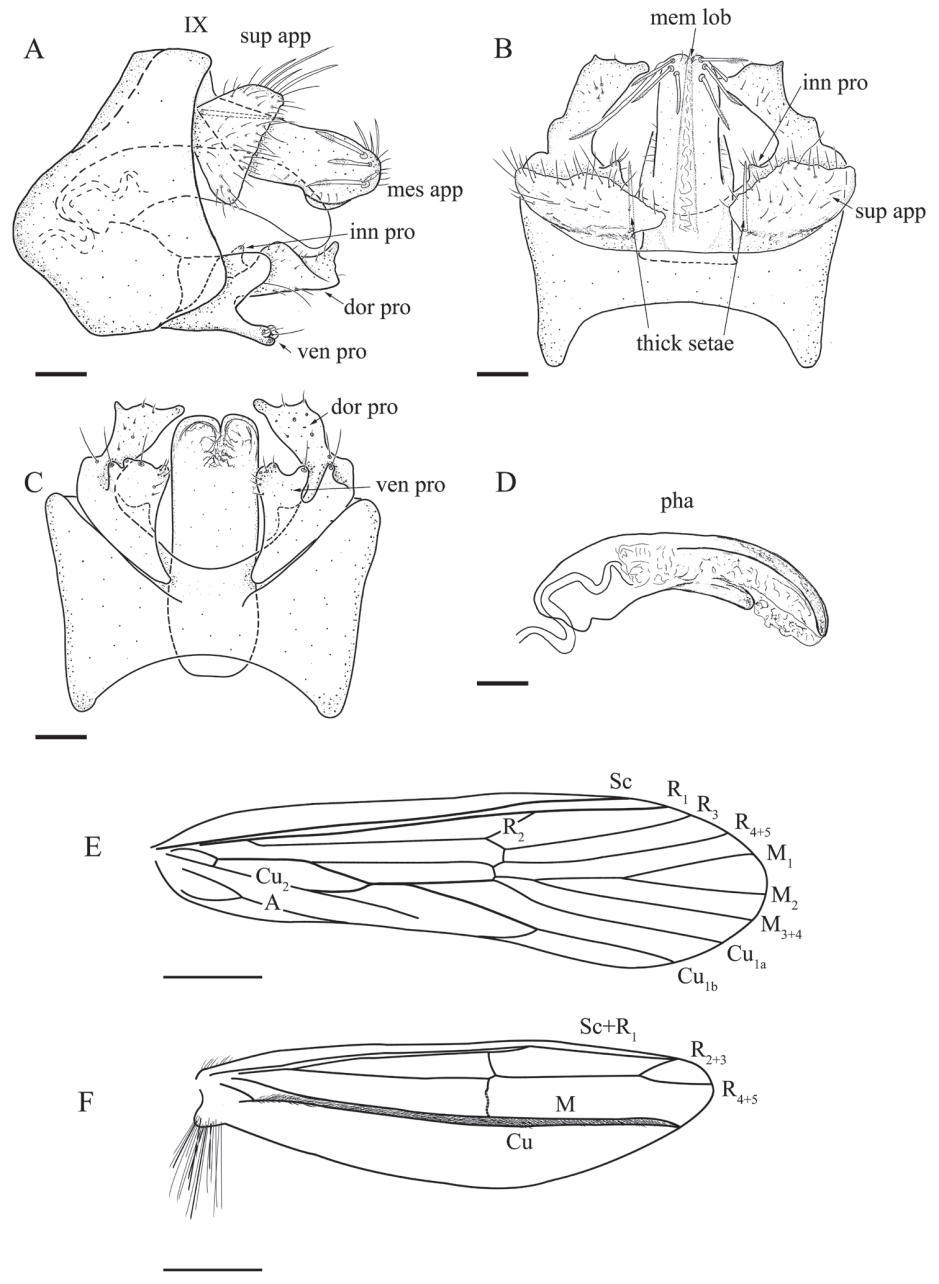
*Molannatruncata* sp. nov. Male adult, holotype **A** genitalia, lateral view **B** genitalia, dorsal view **C** genitalia, ventral view **D** phallus, lateral view **E** forewing **F** hind wing. Abbreviations: sup app, superior appendage. mes app, mesal appendage; mem lob, membranous lobe; dor pro, ven pro, inn pro, dorsal, ventral, and inner processes of inferior appendage, respectively; pha, phallus. Sc, Subcosta; R, Radius; M, Media; Cu, Cunitus; A, Anal. Scale bars: 200 μm.

**Figure 2.**
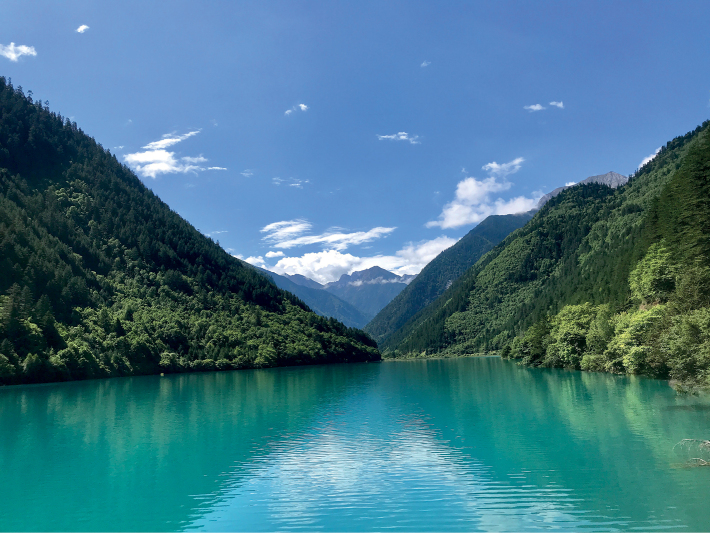
Type locality of *Molannatruncata* sp. nov., Xi-niu-hai in the Jiu-zhaigou National Nature Reserve, China.

##### Other specimens.

1♂ P.R. China, Si-chuan Province, Aba Prefecture, Jiu-zhaigou County, Jiu-zhaigou National Nature Reserve, Wu-hua-hai, 33°9'32"N, 103°52'55"E, alt. 2377 m, 20 VII 2014, leg. Y. Cao (NJAU). 2♂ P.R. China, Si-chuan Province, Aba Prefecture, Jiu-zhaigou County, Jiu-zhaigou National Nature Reserve, Lu-wei-hai, 33°13'18"N, 103°54'48"E, alt. 2299 m, 10 VII 2019, 19 VII 2014, leg. C.Y. Qin (NJAU).

##### Diagnosis.

The new species is similar to *M.yaeyamensis*Ito 2006 from Japan (Ishigaki and Iriomote islands), but can be differentiated by the following characters: (1) superior appendages in *M.truncata* sp. nov. in dorsal view have subrhomboid shape and basally bearing a slender thick seta, whereas *M.yaeyamensis* have subsquare superior appendages with no such thick setae in dorsal view; (2) in *M.truncata* sp. nov. mesal appendages have a subrectangular shape in lateral view, whereas in *M.yaeyamensis* mesal appendages have an ellipsoidal shape in lateral view; (3) inferior appendages with tiny triangular inner processes in *M.truncata* sp. nov., compared to long digitate inner processes (about 2/3 as long as dorsal process) in *M.yaeyamensis*; and 4) ventral processes of inferior appendages in *M.truncata* sp. nov. in ventral view are somewhat stub-like and the lateral margin has a distally bristled, tiny process, which is both absent in *M.yaeyamensis*.

##### Description.

Specimens in alcohol with compound eyes black, thorax, abdomen and legs black to grayish white, without patterns. Body medium-sized, length 7.3–7.7 mm (*N* = 3). Head 0.95 mm long, about 1.75 times wider than length, ocelli absent. Front of vertex with subquadrate setal wart, posterolateral portion with two pairs of setal warts. Pronotum nearly trapezoidal, Pronotum anterior margin slightly sinuous, slightly concave anteromesad, posterior margin slightly concave, with one pair of setal warts.

***Male genitalia***: Abdominal segment IX in lateral view (Fig. 1A), irregularly pentagonal, convex anteriorly and posteriorly. Superior appendages in lateral view (Fig. 1A), trapezoidal, covered with many long and short setae, posterior margin straight; in dorsal view (Fig. 1B), subrhomboid, posterior margin irregularly serrated, each with slender thick setae at ventromesal base. In lateral view (Fig. 1A) mesal appendages subrectangular shape with narrow base and wider distal part, distal end slightly produced ventrad, with each side having 3–4 thick long setae, setose apically; in dorsal view, somewhat tubular, with base slightly thickened, with longitudinal membranous lobe mesally from base to apex. In lateral view, inferior appendages one-segmented, slightly shorter than mesal appendages, each divided into dorsal and ventral processes externally, and with a tiny triangular inner process (Fig. 1B); dorsal processes each with apex curved upwards in lateral view (Fig. 1B), sparsely setose, apical and lateral margins each sinuate, inner margins each arc-shaped in dorsal view; ventral processes in lateral view halfway shorter than dorsal processes, in ventral view (Fig. 1C) somewhat stub-like, lateral margin with distally bristled, tiny process. Phallus arched, with one pair of thin, long sclerites on apical half of dorsal surface (Fig. 1D).

***Male wings***: Forewings (Fig. 1E): Venation fairly complete, typical for the genus, without obvious marks. R_2_ very short jointed with R_1_, M 3-branched. M and Cu_1_ fused at very base, Cu_2_ not extended to margin, 1A and 2A merged at base and running to posterior margin and then curved anteriad to Cu_1b_. Hind wings (Fig. 1F): Venation of male hind wing much reduced, with band of dark setae running near midline.

##### Etymology.

The Latin adjective *truncatus*, -*a*, *um* refers to the truncate shape of the superior appendages posterior margin in lateral view.

##### Distribution.

China (Si-chuan).

### ﻿Key to the adult males of *Molanna* Curtis, 1834 from Oriental region

**Table:** 

1	In lateral view, mesal appendages distal end furcated	**2**
–	In lateral view, mesal appendages distal end unfurcated	**5**
2	Distal end of mesal appendages dorsum with 3 large spines	***M.gamdaha* (Fig. 3A)**
–	Distal end of mesal appendages dorsum without 3 large spines	**3**
3	In lateral view, superior appendages triangular, with posterior margins concave	***M.crinitaa* (Fig. 3B)**
–	In lateral view, superior appendages trapezoidal	**4**
4	In lateral view, mesal appendages with upper and lower lobes pointing to and nearly contacting each other	***M.saetigera* (Fig. 3C)**
–	In lateral view, mesal appendages with upper and lower lobes divided widely	***M.oglamar* (Fig. 3D)**
5	Inferior appendages without ventral processes	**6**
–	Inferior appendages with ventral processes	**10**
6	Superior appendages in lateral view leaf-shaped or trapezoid	**7**
–	Superior appendages in lateral view finger-shaped	**9**
7	Superior appendages in lateral view, leaf shape	***M.kunmingensis* (Fig. 3E)**
–	Superior appendages in lateral view, trapezoid shape	**8**
8	In lateral view, mesal appendages finger-like	***M.moesta* (Fig. 3F)**
–	In lateral view, mesal appendages inflated, hammer-like	***M.paramoesta* (Fig. 3G)**
9	In lateral view, mesal appendages tapering from base to apex, with distal half curved downwards at 90 degree	***M.taprobane* (Fig. 3H)**
–	In lateral view, mesal appendages not as above	***M.xiaguana* (Fig. 3I)**
10	Inferior appendages without inner processes	**11**
–	Inferior appendages with inner processes	**12**
11	Ventral processes of inferior appendage with thorn distally	***M.jolandae* (Fig. 3J)**
–	Ventral processes of inferior appendage without thorn distally	***M.cupripennis* (Fig. 3K)**
12	Inferior appendages with tiny triangular inner processes	***M.truncata* sp. nov. (Fig. 3L)**
–	Inferior appendages with slender inner processes, about 2/3 as long as dorsal process	***M.yaeyamensis* (Fig. 3M)**

**Figure 3.**
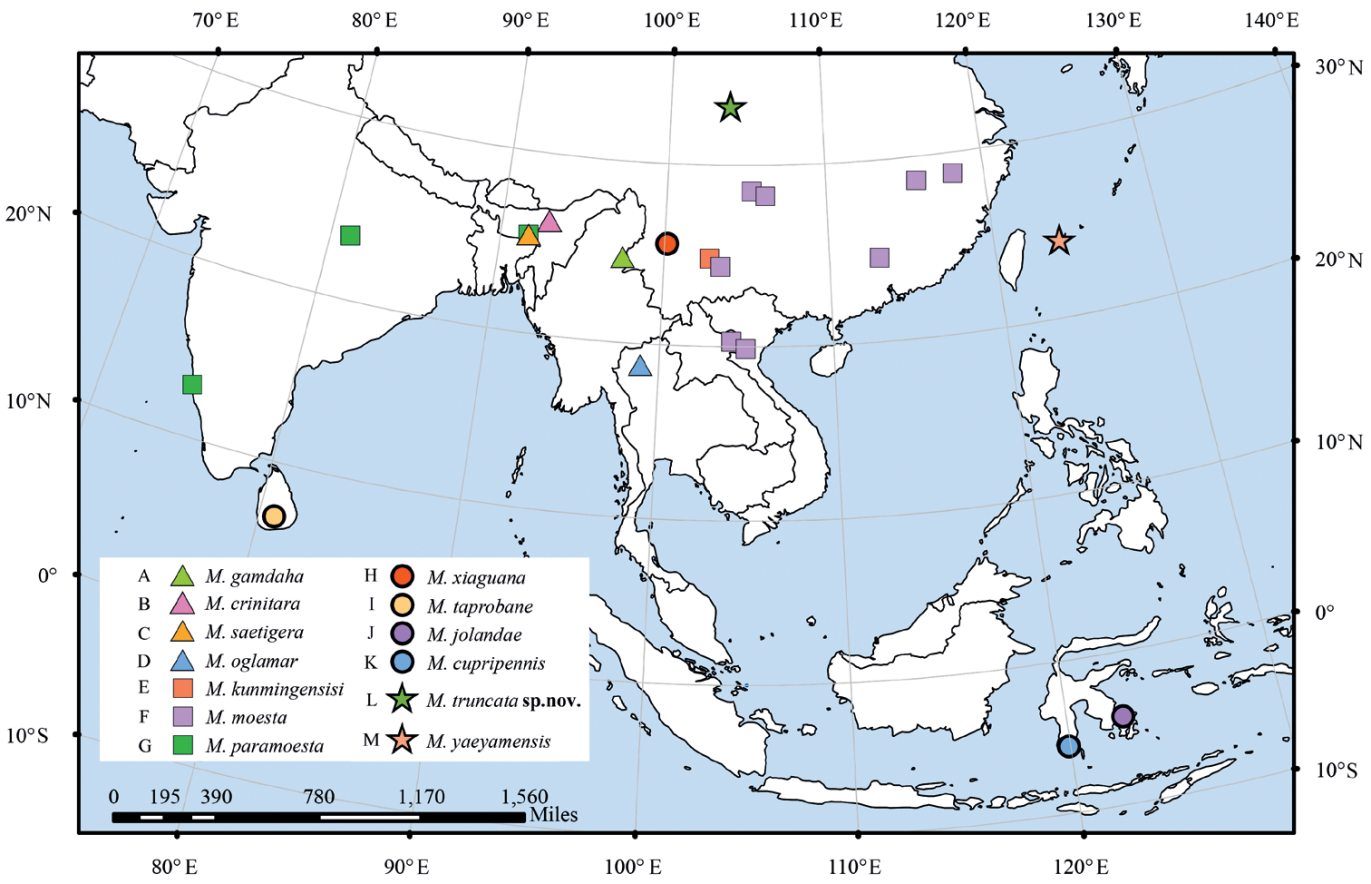
Distribution of *Molanna* species in the Oriental region.

### ﻿DNA barcodes analyses

**Figure 4.**
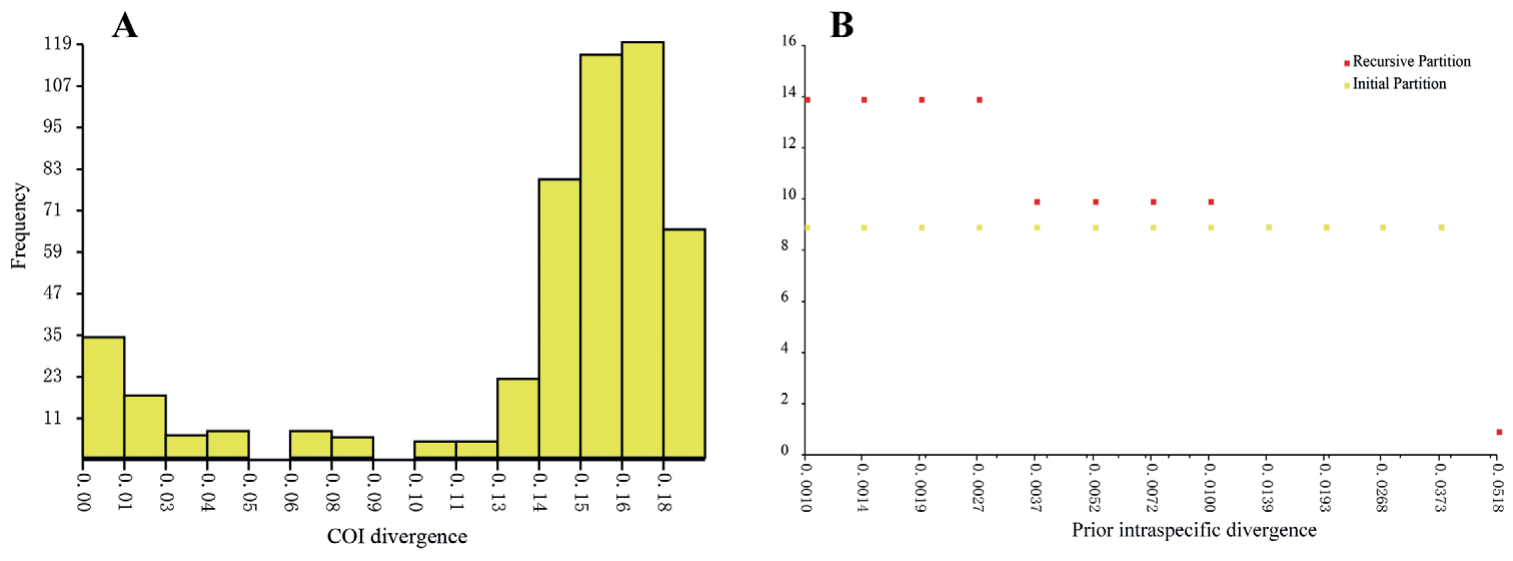
Histogram of pairwise K2P distances and number of the OTUs of 32 aligned sequences **A** the histogram was created using the K2P model in the Automatic Barcode Gap Discovery (ABGD) analysis. The horizontal axis shows the pairwise K2P-distance, and the vertical axis shows the number of pairwise sequence comparisons **B** the number of the OTUs by the prior intraspecific divergence calculated with the ABGD online-tool.

**Figure 5.**
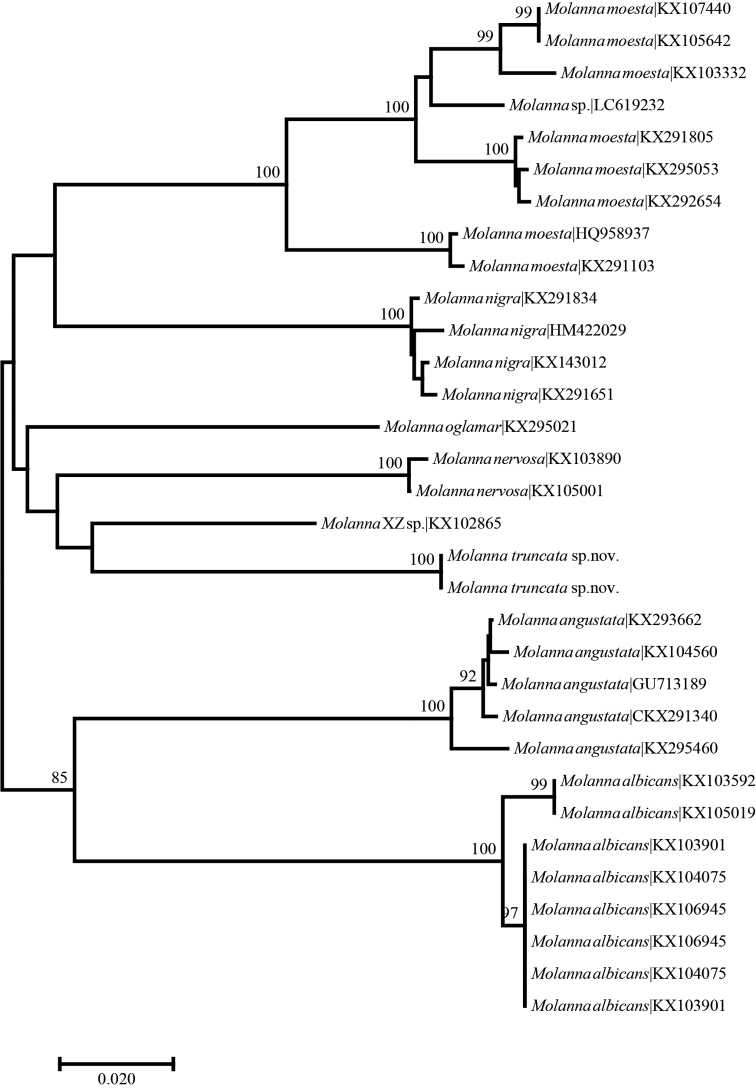
Neighbor-joining cladogram based on the 658 bp long mtCOI sequence of eight *Molanna* species. Numbers on branches represent bootstrap support (>70%) based on 1000 replicates; scale equals K2P genetic distance.

The aligned 32 sequences ranged from 634 to 658 base pairs, including 29 sequences with a full barcode length of 658 base pairs. As some morphospecies showed comparably high intraspecific divergence, no definite “barcode gap” was observed based on pairwise distance (Fig. 4A). The ABGD analysis of the genus *Molanna* recognized 9 OTUs with a prior intraspecific divergence of P_max_ = 3.59% (Fig. 4B). The NJ tree based on 32 COI*Molanna* haplotypes conducted in the current study does not provide clear information on species differentiation, except for *M.angustata* and *M.albicans* (Zetterstedt, 1840) (Fig. 5), both distributed in the Palaearctic region. Even though each of the species was clustered in separate clades, division into clades is not supported by the presented NJ analysis (Fig. 5). However, the NJ analysis suggests relatively high differentiation in populations of *M.moesta* (Fig. 5). Two *Molannatruncata* sp. nov. haplotypes were clustered; however, their differentiation from the “*Molanna* XZ.sp.(KX102865)” haplotype is not supported by the NJ tree. The interspecific divergence (K2P*p* value) ranged from 10.1% to 19.5% (Table 1), with the mean divergence of 16.16%. The lowest intraspecific divergence was observed for haplotypes from European populations of *M.angustata* and *M.nigra* (Zetterstedt, 1840) and *M.albicans*, as well as Russian and Japanese populations of *M.moesta* (Table 1). Highest intraspecific divergence was observed in *M.moesta* (Table 1). The mean intraspecific divergence of all species was 1.58% with a maximum of 8.50% in *M.moesta*. In addition, one unnamed species *M.* sp. ( BOLD:AAP1029) was associated with *M.moesta* clades. Divergence of *M.truncata* nov. sp. haplotypes from all other *Molanna* haplotypes ranged from 10.1% (from the “*Molanna* XZ.sp. haplotype) to 18% (from *M.angustata*, Table 1).

## ﻿Discussion

In order to verify the description of *Molannatruncata* sp. nov., we compared the illustrations of male genitalia of all available species and wing venations of most species (Curtis 1834; Banks 1906; Hwang 1957; Wiggins 1968; Malicky and Chantaramongkol 1989; Nebois 1993; Malicky 1994; Ito 1998, 2006; Oláh and Johanson 2010). The male genitalia varied greatly among *Molanna* species in the superior appendages, the mesal appendages and in the inferior appendages. However, some species showed similarities in these characters, as well as in the hindwing venation. Considering the above-mentioned similarities and differences in morphology, we could argue for particular groups of species within the Oriental *Molanna* species.

The first group is characterised by the superior appendages with the height at least twice as long as its length or approximately equal to its length, and the media veins unbranched or occasionally 2-branched in hindwings. This group could be further divided into three subgroups according to morphology of the mesal appendages, inferior appendages and venation degeneration in the hindwings. The first subgroup consists of *M.paramoesta* Wiggins, 1968 and *M.moesta*, in which the posterior margins of the superior appendages are concave, the mesal appendages are oblique and unfurcated at the distal end when viewed laterally; and the inferior appendages are without any ventral processes. Although these two species both have a wide distribution in the Oriental region, but their ranges do not overlap. The second subgroup consists of *M.gamdaha* Oláh & Johanson, 2010, *M.crinita* Wiggins, 1968, *M.saetigera* Wiggins, 1968 and *M.oglamar* Malicky & Chantaramongkol, 1989. In this subgroup, the mesal appendages are furcated at the distal end, the inferior appendages divided into dorsal, ventral and inner processes. Furthermore, media veins are usually unbranched and fused with cubitus at the base or at the distal end. Unlike *M.paramoesta* and *M.moesta*, these four species are regional endemics, also with non-overlapping ranges. The relatively localized dispersal of adults and the disjunct distribution of adequate habitats in some cases lead to small scale allopatric speciation (Vitecek et al. 2015; Thomas et al. 2020). *Molannasaetigera* and *M.paramoesta*, on the contrary, have overlapping ranges. The third subgroup is composed of *M.truncata* sp. nov. and *M.yaeyamensis* in which the mesal appendages are unfurcated at the distal end in lateral view. The inferior appendages and media veins are, however, the same as in the latter group.

The second group is characterised by the digitate superior appendages and the variable mesal appendages, which are either hammer-like or with their distal ends curved ventrad. The hindwings have relatively complete venation. We divided the group into two subgroups based on the shape of inferior appendages. The first subgroup consists of *M.taprobane* Flint, 1973 and *M.xiaguana*, having elongate-triangular inferior appendages. The second subgroup consists of *M.jolandae* Neboiss, 1993 and *M.cupripennis* Ulmer, 1906, with bifurcated inferior appendages. both of which show rather unique distribution patterns in Indonesia (i.e., restricted allopatric distribution on Sulawesi; Fig. 3). They exhibit similar morphology, however, genetic data were not available for the current study, thus their relationship and evolutionary history remain to be investigated. Sulawesi island is however, known as a biodiversity hotspot due to its complex geological history (e.g., Tänzler et al. 2016).

**Figure 6.**
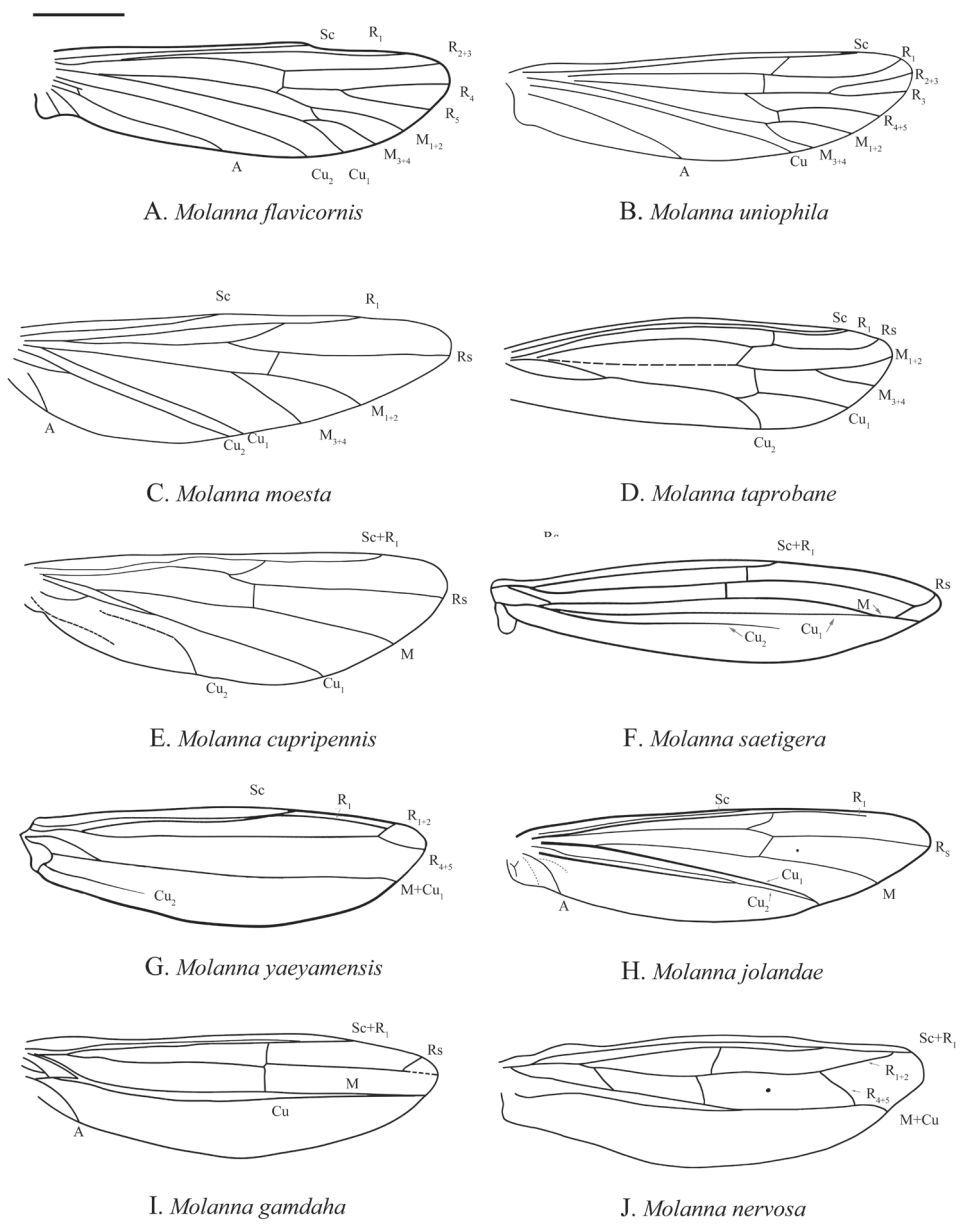
Male hind wings of eight known *Molanna* species. **A***M.flavicornis***B***M.uniophila***C***M.moesta***D***M.taprobane***E***M.cupripennis***F***M.saetigera***G***M.yaeyamensis***H***M.jolandae***I***M.nervosa***J***M.gamdaha*. Scale bar: 200 μm.

**Figure 7.**
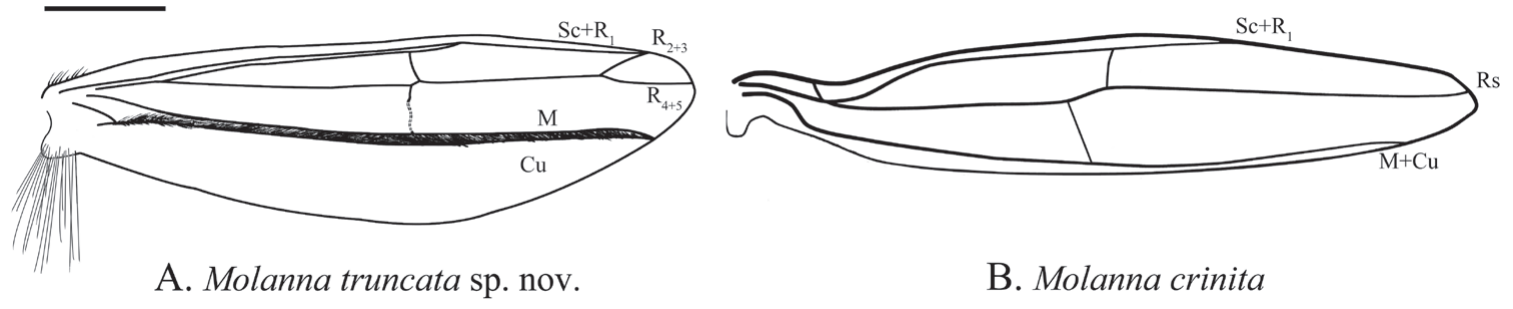
Male hind wings of *Molannatruncata* sp. nov. and *M.crinita*. **A***M.truncata* sp. nov. **B***M.crinita*. Scale bar: 200 μm.

Wings are one of the most important organs of insects, and venation modifications may reflect successful adaptation to different environmental conditions. Based on Schmid’s terminology (Schmid 1998) for the hindwing venation of male *M.flavicornis*, we compared the venation of nine known males of *Molanna*, and found that venation showed a trend of degradation. *Molannaflavicornis* and *M.uniophila* Vorhies, 1909 had the most complete venation in the hindwing, i.e., subcosta was free, radius had 4 branches, media had 2 branches, and cubitus 2 branches. The hindwing venation of these two species could represent the primitive form (Fig. 6A, B). Other species showed venation of hindwings more or less reduced (Figs 6C–J, 7A, B). Regarding the evolution of hindwings venation in the genus, further support from molecular data could help clarify the true evolutionary pattern.

In previous barcode studies on Trichoptera, thresholds of intraspecific divergence (as uncorrected pairwise differences in the COI region) have been estimated to reach at most 11.7% in Hydropsychidae Curtis, 1835 (Zhou et al. 2007) and 11% in *Drusus* Stephens, 1837 (Kučinić et al. 2016). In this study, the ABGD analysis of *Molanna* inferred 9 OTUs and thresholds of interspecific divergence of 10%; however, it seems that *M.moesta* has diverged into three geographic populations (*M.moesta* from Laos, Russia and Japan were recognized as two OTUs; Table 1), with a threshold of intraspecific divergence ranging between 0.30%–8.50% (Table 1). *Molannatruncata* sp. nov. is morphologically most similar to *M.yaeyamensis*, but molecular data for the latter were not available. The minimum interspecific divergence was 10.10% between *M.truncata* sp. nov. and *M.* XZ sp. As the *M.* XZ_sp. was collected in close proximity to the known range of *M.yaeyamensis*, it is highly likely that *M.* XZ_sp. is indeed *M.yaeyamensis*. However, in order to check this, we would need to examine the specimen in detail. The suggested clustering of Oriental *Molanna* into groups and subgroups based on the structure of male genitalia and the hindwing venation should be further evaluated using an integrated approach, i.e., more detailed morphological analysis encompassing more specimens, a multigene phylogeny including all *Molanna* species and detailed species distribution (Johanson et al. 2012; Jiang et al. 2021). Such an approach would enable reconstruction of the history of the genus in the Oriental region; however, at present, data are not available.

*Molannatruncata* sp. nov. is endemic to Jiuzhaigou Natural Reserve. Apart from this species, we have collected an additional 24 species of Trichoptera (belonging to 22 genera and 14 families) during 2014–2019 in the Reserve (Cao et al. 2016), however, it is the only *Molanna* species occurring in the area. The area harbours high diversity of freshwater habitats (114 alpine lakes, 17 groups of waterfalls, 47 springs, and 11 sections of rapids; Deng 2012), thus, high diversity of Trichoptera can be expected. The Reserve is an example of typical karst geology, with a high amount of travertine calcite deposits in freshwater habitats (Wang et al. 2018). The majority of the lakes are oligotrophic, with low concentrations of total suspended solids and low turbidity (Li et al. 2020). Water temperature is relatively low in the lakes, whereas conductivity, alkalinity, and pH are relatively high (Cao et al. 2016). Adults of *M.truncata* sp. nov. were collected from one of such alpine lakes located at altitudes ranging from 2299 to 2377 m.

## Supplementary Material

XML Treatment for Molanna truncata
